# Relationship between the Quality of Service Provided through Store-and-Forward Telemedicine Consultations and the Difficulty of the Cases – Implications for Long-Term Quality Assurance

**DOI:** 10.3389/fpubh.2015.00217

**Published:** 2015-09-25

**Authors:** Richard Wootton, Joanne Liu, Laurent Bonnardot

**Affiliations:** ^1^Norwegian Centre for Integrated Care and Telemedicine, University Hospital of North Norway, Tromsø, Norway; ^2^Faculty of Health Sciences, University of Tromsø, Tromsø, Norway; ^3^MSF International, Geneva, Switzerland; ^4^Department of Medical Ethics and Legal Medicine, Paris Descartes University, Paris, France; ^5^Fondation Médecins Sans Frontières, Paris, France

**Keywords:** telemedicine, telehealth, quality assurance, quality control, LMICs

## Abstract

We examined the difficulty of telemedicine cases and the quality of the resultant consultation in a mature store-and-forward telemedicine network. A random sample of 10 telemedicine cases was selected from those occurring over a 3-month period (5% of the workload) and they were scored by three experienced observers. Inter-observer agreement on the difficulty scores was poor (Fleiss’s kappa = 0.18) and it was also poor on the consultation quality scores (Fleiss’s kappa = 0.11). Differences between observers were minimized by consensus scoring, and the cases were re-assessed jointly by two observers. Based on the consensus scores, there was a weak negative relation between output quality and case difficulty, i.e., the more difficult cases tended to result in lower quality consultations. However, the effect was non-significant (*P* = 0.59) and a larger study might be helpful. In the meantime, routine monitoring of telemedicine service quality will continue in the interests of quality assurance. As yet, there is no evidence on which to base a correction for case difficulty.

## Introduction

Médecins Sans Frontières (MSF), a non-governmental humanitarian medical organization, operates a store-and-forward tele-expertise network to support its field staff (1). This service has been in operation for 6 years and has managed approximately 2000 telemedicine cases; it can, therefore, be considered reasonably mature. In operating any routine healthcare delivery service, those responsible will wish to monitor it using quality assurance (QA) principles. In the case of the telemedicine network, the QA work would concern long-term monitoring with the aim of detecting reductions in its performance and then correcting them. This requires a method of measuring the output of the network, which we have previously defined as the *quality* of the teleconsultations being produced. We have proposed a method for measuring quality and demonstrated its feasibility (2).

However, in real life each telemedicine case is different: some will be easy to manage by telemedicine and some will be difficult. The difficulty of an individual case will depend on four main factors (3):

1. the description of the problem
2. the complexity of the patient
3. the availability of network resources for providing an answer
4. the availability of resources for implementing the advice (e.g., for making a diagnosis or for providing treatment).

The latter point refers to the patient’s environment and constitutes an important dimension since any management must depend on the patient’s environment. Taking account of the environment is often challenging because there are multiple factors involved (4):

- the complexity of the situation, for example, due to political, cultural, socioeconomic, or environmental factors
- the health care system available, such as the infrastructure, organization, and human resources
- the characteristics of the health workers who are managing the patient, such as their background, competencies, and experience.

To facilitate the operation of the MSF telemedicine system, information is available to the specialists concerning both the referrer and the patient’s health care facility. This allows the specialist to tailor the advice provided to suit the local environment.

We have proposed a method for measuring the difficulty of cases and demonstrated its feasibility (3). The question which then arises is how much the output of the network (consultation quality) is affected by the input (case difficulty). If the effect is substantial, then it might be desirable to allow for case difficulty in the long-term monitoring of output quality.

The aim of the present study was to investigate the relation between consultation quality and case difficulty.

## Methods

The relation between output quality and input difficulty was investigated in a sample of cases from the MSF telemedicine network:

1. a random sample of 10 telemedicine cases was selected from those occurring over a 3-month period (the first 3 months of 2015)
2. assessments of these cases were made independently by three experienced observers. Case difficulty was scored by answering 17 multiple choice questions (no/perhaps/yes), resulting in a score from 0 = very easy to 10 = very difficult (3). Consultation quality was scored by answering 11 multiple choice questions (no/perhaps/yes), resulting in an overall quality score from 0 = very poor to 10 = very good (2). For convenience, the questions are reproduced in the Supplementary Material
3. agreement between observers was measured for difficulty scores and for quality scores using Fleiss’s kappa statistic
4. the cases were also assessed jointly by two observers to obtain consensus values for the scores. Several conference calls were used to discuss cases and reach the consensus
5. the relation between consultation quality and case difficulty was examined by regression analysis.

Ethics permission was not required because patient consent to access the data had been obtained and the work was a retrospective chart review conducted by the organization’s staff in accordance with its research policies.

## Results

During the 3-month period, the telemedicine network dealt with 185 clinical cases. The random sample of 10 cases, therefore, represented 5.4% of the caseload. Brief details are provided in Table 1.

**Table 1:** **Details of the 10 randomly selected cases**.

Case no.	Patient	Location	Type of query	Specialist(s) answering	Time to first reply (days)
1620	Female, 6 years	South Sudan	Infectious disease	Pediatrician	0.2
1587	Male, 4 years	Papua New Guinea	Radiology (exclude TB)	Radiologist	0.4
1569	Female, 18 years	Afghanistan	Infectious disease	Infectious disease specialist	0.1
1561	Female, 79 years	Cambodia	Radiology (lung cancer)	Radiologist	0.3
1693	Male, 6 years	DR Congo	Radiology (pyomyositis)	Radiologist	1.0
1676	Male, 14 months	DR Congo	Radiology (pneumonia)	Radiologist	0.2
1584	Female, 58 years	Syria	Neurology	Neurologist	1.0
1576	Male, 7 years	South Sudan	Ophthalmology	Pediatrician; ophthalmologists (2)	0.8
1586	Male, 4 years	Papua New Guinea	Radiology (TB treatment)	Radiologist	0.4
1695	Female, 9 years	DR Congo	Radiology (pneumothorax)	Radiologist	0.2

The sample of cases was assessed independently by a panel of three observers (Table 2). The correlation between output quality and case difficulty was not significant for any observer considered individually. However, inter-observer agreement on the difficulty scores was poor (Fleiss’s kappa = 0.18) and it was also poor on the consultation quality scores (Fleiss’s kappa = 0.11).

**Table 2:** **Details of the observers**.

Observer	Background	No. of queries managed^a^	Percentage of total queries managed^a^
1	Experienced MSF clinician. Several field placements	533	21
2	Experienced MSF clinician. Several field placements	510	20
3	Telemedicine researcher	844	33

*^a^From April 2010 to March 2015*.

**Figure 1:**
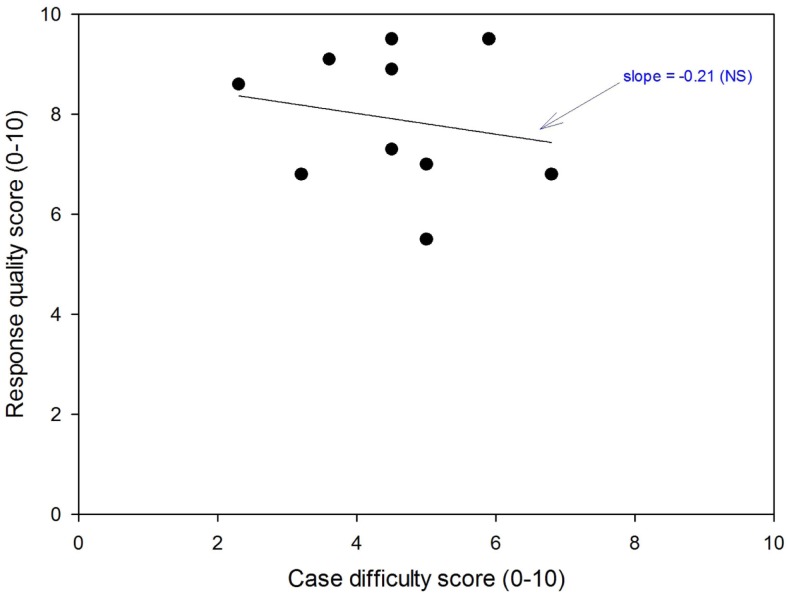
**Relation between the difficulty of the telemedicine cases and the quality of the subsequent teleconsultation**.

The cases were then re-assessed jointly by two observers, who discussed each scoring disagreement and came to a consensus. Based on the consensus scores, there was a weak negative relation between output quality and case difficulty, i.e., the more difficult cases tended to result in lower quality consultations, see Figure 1. However, the effect was not significant (*t* = 0.56, *P* = 0.59).

## Discussion

When telemedicine cases were assessed independently by three observers, the inter-observer agreement on the scores was poor. This was probably due to differences in the observers’ background (one observer had no MSF field experience) and to the absence of formal training materials about the two scoring systems used. The value of prior training for an expert panel which is undertaking quality assessment (using training seminars and teaching manuals, for example) has been shown to be important in ensuring valid estimates (5).

From the consensus sessions (after scoring cases independently), some points emerged which could improve the quality of future scoring:

1. questions need to be defined precisely, so that there can be no doubt about the subject of the question; detailed guidance may be necessary to clarify each question. For example, both the quality and the difficulty scores contained a question about whether sufficient information had been provided by the referrer. In some of the cases which were assessed, we observed that the specialists involved in the cases had started their responses by asking a question. It seems natural to assume that if the specialist begins by asking a question, the referrer cannot have provided sufficient information in the original referral
2. observers who assess a case that refers to their own specialty tend to be more demanding in their scoring
3. observers sometimes change their mind when re-scoring a case. This may be due to a lack of attention initially, or to a change of mind after hearing the opinion of other observers.

Using consensus scores between two observers showed that there was a weak negative relation between output quality and case difficulty, i.e., the more difficult cases tended to result in lower quality teleconsultations. Why does this matter?

### Relation between consultation quality and case difficulty

**Figure 2:**
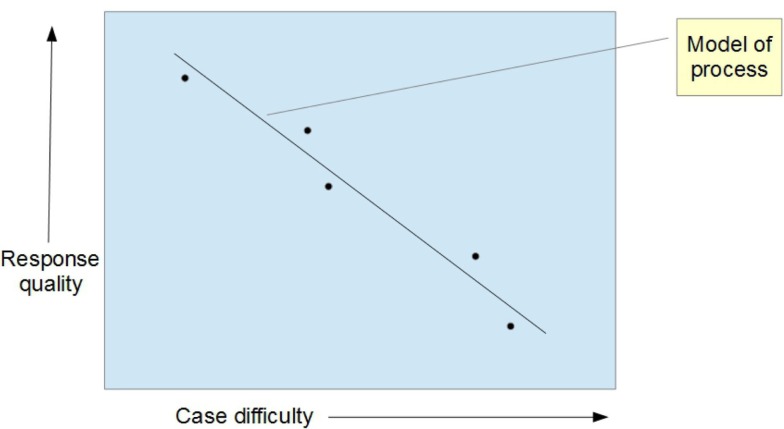
**Relation between the difficulty of the case and the quality of the response produced by the network**.

If the input and output of a telemedicine network (i.e., case difficulty and consultation quality) can be measured, then the process itself can be quantified; this is analogous to the transfer function describing the behavior of a black box model. Measuring the transfer function can be done using a sample of cases that cover a range of input values (Figure 2). The best descriptor of the process can be established from the observations which have been made by regression analysis. Note, however, that each variable has an observational error (since each will be estimates from a panel of observers). So techniques for regression with errors in both *Y* and *X* are required, i.e., this is not the standard regression situation.

Also note that the transfer function must be established quickly enough that the underlying process can be assumed to be stationary. In the present study, 10 cases were randomly selected as being about the maximum number that could be analyzed, given the practical constraints on the observers. Obviously, the smaller the sample, the less likely it is that the stationarity assumption would be violated. Thus, the number of cases sampled represents a compromise.

**Figure 3:**
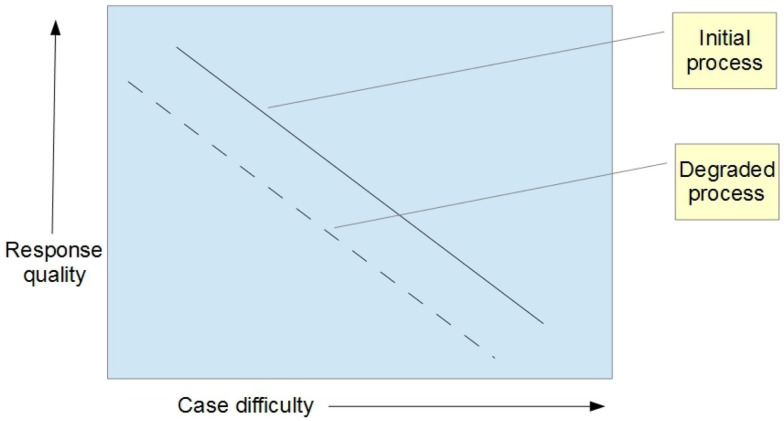
**Change in the process (degradation) from baseline**.

Once the baseline transfer function has been established, it can be used to detect changes in the behavior of the network. For example, the transfer function analysis could be repeated after about 6 months, and compared with the baseline (Figure 3).

**Figure 4:**
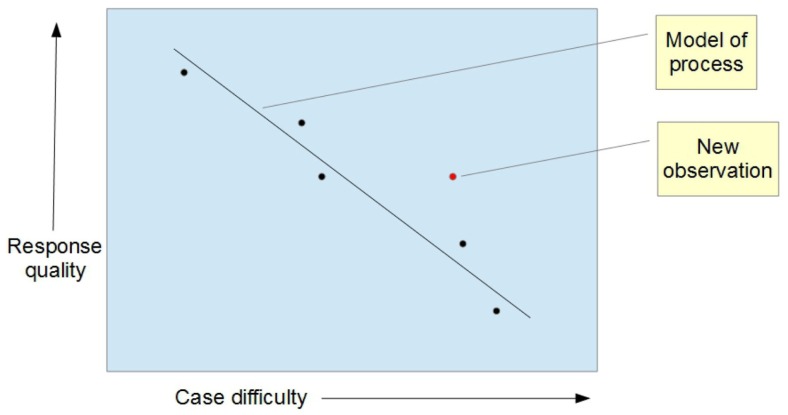
**Adding a new point: is it different from the model**?

Alternatively, we could add individual observations once a week, say. To detect a change, each new point would be examined to see if it was significantly different from the model (Figure 4). If not, it would be added to the model. If it was different, then it could be inferred that the process had changed.

### Strengths and weaknesses of the study

We are not aware of previous work on the relation between the difficulty of cases in a telemedicine network (of any kind) and the value of the resulting consultation. One strength of the study was that it was performed using real cases, selected at random from a mature telemedicine network. Another strength was that the observers who carried out the assessments were experienced in operating the telemedicine network: between the three of them, they had handled almost three-quarters of the cases on the network in the first 6 years (Table 2).

On the other hand, the scoring system used in the present work rested ultimately on the subjective judgments made by the observers. As was clear from their independent assessments, their agreement was poor – something that would have been improved by prior training – so consensus scoring was used to eliminate inter-observer differences. One of the observers had previously managed some of the randomly selected cases, so there is a possibility of unconscious bias in his scoring, although given the 6-month interval between case management and scoring that seems unlikely. Another weakness was that the study was carried out using a small sample, and it is conceivable that a Type 2 error may have been made. Nonetheless, the results show that while a statistical relation between the output quality and the input case difficulty may exist, the magnitude of the effect is small. We, thus, feel confident in ignoring it in future long-term monitoring of network performance.

## Conclusion

The present study examined the difficulty of telemedicine cases and the quality of the resultant consultation provided at distance. Differences between observers were minimized by consensus scoring, and it appears that use of a scoring manual would be important in minimizing inter-observer differences in future. The results suggest that more difficult cases tend to result in lower quality teleconsultations, although the effect is non-significant. The study was based on a small sample and a larger study might be helpful. In the meantime, routine monitoring of telemedicine service quality will continue in the interests of QA. As yet, there is no evidence on which to base a correction for case difficulty.

## Conflict of Interest Statement

The authors declare that the research was conducted in the absence of any commercial or financial relationships that could be construed as a potential conflict of interest.

## Supplementary Material

The Supplementary Material for this article can be found online at http://journal.frontiersin.org/article/10.3389/fpubh.2015.00217
