# Supplementary material for: Relationship between the Quality of Service Provided through Store-and-Forward Telemedicine Consultations and the Difficulty of the Cases – Implications for Long-Term Quality Assurance
Source: Front Public Health. 2015 Sep 25;3:217. doi: 10.3389/fpubh.2015.00217 (PMC4585167; doi:10.3389/fpubh.2015.00217)
Supplement: Supplementary file 1 [file Data_Sheet_1.PDF]

Appendix. Questions (reproduced with permission of Frontiers from previous publications[2,3]).

A. Questions used to assess case difficulty. The questions were scored on a three-point scale (1=no, 2=perhaps; 3=yes; and 0=don't know) with the high end of the scale indicating less difficulty. Note that scoring for certain questions was reversed (e.g. How severely ill was the patient?) where a response at the high end of the scale indicated more difficulty. For convenience, the resulting total scores were transformed to lie in the range 0-10, using the transformation: (n - min) x 10 / (max - min), where n represents the total score for the case concerned, min represents the lowest score possible and max represent the highest score possible.

**Question**	**Response**
1. How well formulated was the question?	very poor/acceptable/excellent
2. Was the information provided satisfactory? (including, if appropriate, any images and their quality)	no/perhaps/yes
3. How severely ill was the patient?	not very/moderately/very
4. Were there multiple co-occurring medical conditions?	no/perhaps/yes
5. Was it difficult to determine an accurate diagnosis? (e.g. the conditions were poorly differentiated and the symptoms were unrecognised or not identifiable)	not very/moderately/very
6. What was the degree of impairment or disability of the patient?	not impaired/moderate impairment/very impaired
7. What was the level of need for comprehensive care management?	none/moderate/high
8. Was the care-coordinator resource available promptly and with the right experience/expertise to handle the case? (if manual allocation was being used)	no/perhaps/yes
9. Was the required specialist(s)/subspecialist(s) available?	no/perhaps/yes
10. Did the referral site have satisfactory resources for treatment locally?	no/perhaps/yes
11. Was it possible to transfer patients for specialist treatment elsewhere?	no/perhaps/yes

B. Questions used to assess the quality of the teleconsultation. The questions were scored on a three-point scale (1=no, 2=perhaps; 3=yes; and 0=don't know) with the high end of the scale indicating higher quality. Note that scoring for certain questions was reversed (e.g. Overall, could the referral have been improved?) where a response at the high end of the scale indicated lower quality. For convenience, the resulting total scores were transformed to lie in the range 0-10, using the transformation described above.

**Question**	**Response choices**
1. Was the question asked by the referring doctor clear?	no/perhaps/yes
2. Did the referrer provide sufficient information?	no/perhaps/yes
3. Were any images provided?	yes/no
4. ... if YES, were the images adequate?	no/perhaps/yes
5. ... if NO, would some images have helped?	no/perhaps/yes
6. Overall, could the referral have been improved?	no/perhaps/yes
7. Was the case sent to an appropriate expert?	no/perhaps/yes
8. Was the answer provided sufficiently quickly?	no/perhaps/yes
9. Was the answer(s) well-adapted for the local environment?	no/perhaps/yes
10. Overall, could the answer have been improved?	no/perhaps/yes
11. Did the telemedicine advice clarify the diagnosis for the doctor and patient?	no/perhaps/yes

12. Did the suggested action help the doctor manage the patient?	no/perhaps/yes
13. Do you think that the eventual clinical outcome will be beneficial for the patient?	no/perhaps/yes
14. Was the consultation useful for the doctors concerned?	no/perhaps/yes
15. Could the allocation/coordination have been improved?	no/perhaps/yes
16. Was the consultation good from the organization's point of view?	no/perhaps/yes
17 Do you have any comments about this case?	(free text)
